# Magnetic Resonance Imaging Radiomics-Driven Artificial Neural Network Model for Advanced Glioma Grading Assessment

**DOI:** 10.3390/medicina61061034

**Published:** 2025-06-03

**Authors:** Yan Qin, Wei You, Yulong Wang, Yu Zhang, Zhijie Xu, Qingling Li, Yuelong Zhao, Zhiwei Mou, Yitao Mao

**Affiliations:** 1 Department of Radiology, Xiangya Hospital, Central South University, Changsha 410008, China; 2 National Clinical Research Center for Geriatric Disorders, Xiangya Hospital, Central South University, Changsha 410008, China; 3 Communication Sciences and Disorders, Oklahoma State University, Stillwater, OK 74075, USA; 4 Department of Pathology, Xiangya Hospital, Central South University, Changsha 410008, China; 5 School of Computer Science and Engineering, South China University of Technology, Guangzhou 510640, China; 6 Department of Rehabilitation, The First Affiliated Hospital of Jinan University, Guangzhou 510630, China

**Keywords:** MRI, radiomics, glioma, grading, artificial neural network model

## Abstract

*Background and Objectives*: Gliomas are characterized by high disability rates, frequent recurrence, and low survival rates, posing a significant threat to human health. Accurate grading of gliomas is crucial for treatment plan selection and prognostic assessment. Previous studies have primarily focused on the binary classification (i.e., high grade vs. low grade) of gliomas. In order to perform the four-grade (grades I, II, III, and IV) glioma classification preoperatively, we constructed an artificial neural network (ANN) model using magnetic resonance imaging data. *Materials and Methods*: We reviewed and included patients with gliomas who underwent preoperative MRI examinations. Radiomics features were derived from contrast-enhanced T1-weighted images (CE-T_1_WI) using Pyradiomics and were selected based on their Spearman’s rank correlation with glioma grades. We developed an ANN model to classify the four pathological grades of glioma, assigning training and validation sets at a 3:1 ratio. A diagnostic confusion matrix was employed to demonstrate the model’s diagnostic performance intuitively. *Results*: Among the 362-patient cohort, the ANN model’s diagnostic performance plateaued after incorporating the first 19 of the 530 extracted radiomic features. At this point, the average overall diagnostic accuracy ratings for the training and validation sets were 91.28% and 87.04%, respectively, with corresponding coefficients of variation (CVs) of 0.0190 and 0.0272. The diagnostic accuracies for grades I, II, III, and IV in the training set were 91.9%, 89.9%, 92.1%, and 90.7%, respectively. The diagnostic accuracies for grades I, II, III, and IV in the validation set were 88.7%, 87.1%, 86.5%, and 86.9%, respectively. *Conclusions*: The MRI radiomics-based ANN model shows promising potential for the four-type classification of glioma grading, offering an objective and noninvasive method for more refined glioma grading. This model could aid in clinical decision making regarding the treatment of patients with various grades of gliomas.

## 1. Introduction

Glioma is the most prevalent primary malignant tumor affecting the central nervous system (CNS) in adults [1], with an annual incidence rate of approximately 6 cases per 100,000 individuals worldwide. Men are 1.6 times more likely to be diagnosed with gliomas than women [2,3]. In the United States alone, an estimated 20,000 new glioma diagnoses occur annually (https://www.nccn.org/patients/guidelines/content/PDF/Brain-Gliomas-Chinese-app.pdf) (accessed on 22 November 2021). Characterized by high disability rates, frequent recurrence, and low survival rates, glioma stands as a global refractory tumor, posing a significant threat to human health.

The grading of CNS tumors serves as an independent risk factor for glioma, significantly impacting the development of treatment strategies, response monitoring, and prognosis assessment. A notable change in the 2021 WHO CNS5 tumor grading involved the classification of neoplasms within tumor types, rather than across different tumor varieties [4]. Tumors are graded on a scale of 1 to 4, primarily based on the resemblance of cancer cells to normal cells. Grade 1 gliomas, which grow slowly and may be relatively benign, generally allow patients to enjoy an extended lifespan, with surgery alone often being a sufficient mode of treatment. Grade 2 glioma cells appear somewhat abnormal and, depending on risk assessment, may or may not necessitate additional therapy following surgical removal [5,6]. Grade 3 cancer cells, which exhibit little similarity to normal cells, proliferate rapidly and invade adjacent tissue. Grade 4 cancer cells, on the other hand, appear highly abnormal, growing and spreading at an accelerated pace. Both grade 3 and 4 tumors require supplementary therapies, such as radiation and chemotherapy, subsequent to surgery [6].

The WHO pathological grade serves as a crucial factor in determining personalized treatment plans and prognoses for glioma patients. As a result, accurately grading gliomas pathologically prior to surgery is of paramount importance. Currently, the gold standard for glioma pathological grading is stereotactic-guided needle biopsy [7,8]. However, this method is invasive and carries risks of infection, intracerebral hemorrhage, and metastasis [9]. Consequently, developing a noninvasive, repeatable method for accurately predicting a glioma’s pathological grade before surgery is of immense scientific and clinical value.

Magnetic resonance imaging (MRI) has been employed for the preoperative pathological grading of gliomas due to its advantages of high soft tissue resolution, exceptional image contrast, and metabolic function imaging, as well as being noninvasive and nonradiative [10]. Nevertheless, imaging features of gliomas with varying pathological grades can intersect and partially overlap, particularly between grades II and III. For instance, contrast enhancement is predominantly characteristic of high-grade gliomas; however, some low-grade gliomas may also exhibit enhancement [11,12]. Moreover, routine imaging diagnosis can be highly subjective, as it depends on the experience of the radiologists involved. These challenges hinder the application of conventional MRI in the accurate preoperative grading of glioma.

As precision medicine emerges as a significant trend in medical development, radiomics has gained prominence. Radiomics is a research methodology that utilizes automated algorithms to analyze medical images of interest, employing various statistical analyses and data mining techniques to extract and unveil key information that is ultimately used for disease diagnosis, classification, and grading [13,14,15]. The majority of prior studies have concentrated on the binary classification of glioma, specifically distinguishing between high-grade and low-grade tumors.

In recent years, artificial neural networks (ANNs) have become a popular research topic within the field of artificial intelligence. ANNs are information processing systems that mimic the functionality and mechanisms of neural networks in the brain. As research on artificial neural networks has advanced, they have been extensively applied and developed in various fields such as pattern recognition, intelligent robotics, medicine, and economics. ANNs have successfully tackled numerous challenging problems and demonstrated exceptional classification efficiency [16,17,18]. To our knowledge, most previous studies on preoperative glioma grading focused on two-grade classification (low-grade glioma and high-grade glioma). For instance, Ding et al. [19] proposed a deep learning and radiomic model to differentiate low-grade glioma (LGG) and high-grade glioma (HGG), achieving satisfactory classification results. However, there have not been reports of a four-grade glioma classification (i.e., grades I, II, III, and IV) approach based on radiomics. To address this gap, we employed an artificial neural network model to noninvasively predict glioma grading using radiomics features extracted from post-contrast-enhanced MR images. We hope that our approach will contribute positively to addressing the clinical challenge of accurately grading gliomas preoperatively.

## 2. Materials and Methods

### 2.1. Patients

From January 2017 to June 2024, we retrospectively reviewed and included 362 patients who met the following criteria in this study: (1) newly diagnosed; (2) complete and satisfactory preoperative MR images that passed quality control; (3) obtainable pathological grade (grades I–IV). Patient demographic information was extracted from our institutional database. This retrospective study received approval from the Medical Ethics Review Committee of Xiangya Hospital Central South University (ethics approval number: 2022111310). Informed consent was waived due to the study’s retrospective nature. The paraffin-embedded surgical specimens were re-evaluated by two experienced pathologists at our institution, each with over eight years of experience in CNS tumor pathological diagnosis. The reassessment was based on the criteria of the 2021 WHO classification of CNS tumors [4]. Both pathologists were blinded to the patients’ information, including radiological and demographic data. Any disagreements were resolved through discussion.

### 2.2. Image Acquisition

All glioma patients underwent MRI examinations. The MRI images were obtained using a 3.0-T scanner (Siemens Healthcare, Erlangen, Germany) equipped with a 64-channel head and a neck receiver coil. The anatomical images encompassed axial T1-weighted images (T_1_WI), T2-weighted images (T_2_WI), fluid-attenuated inversion recovery (FLAIR), and contrast-enhanced T1-weighted images (CE-T_1_WI).

### 2.3. Image Segmentation

Previous studies have shown that contrast-enhanced MR images are the most significant for glioma grading [20,21], As a result, the segmentation of the regions of interest (ROIs) was performed solely on the CE-T_1_WI. Utilizing the ITK-SNAP software (version 3.6.0) (http://www.itksnap.org) (accessed on 10 May 2019), two experienced radiologists (reader 1 and reader 2, each with over 10 years of experience in neuroimaging) manually contoured the tumor boundaries slice by slice. The purpose of involving reader 2 was to obtain the inter-observer (reader 1 vs. reader 2) correlation coefficient. To evaluate the inter-observer (reader 1 vs. reader 2) and intra-observer (reader 1 twice at four-week intervals) correlation coefficients (ICCs), 60 patients were randomly selected. Typically, an ICC of ≥0.75 indicates acceptable reproducibility. Thus, any radiomics feature with an ICC (either inter-observer or intra-observer) of ≤0.75 would be excluded from further analysis. Reader 1 completed the segmentation for the remaining patients. For the 60 randomly selected patients, the first segmentation performed by reader 1 was used for further analysis.

### 2.4. Radiomics Feature Selection

**Figure 1:**
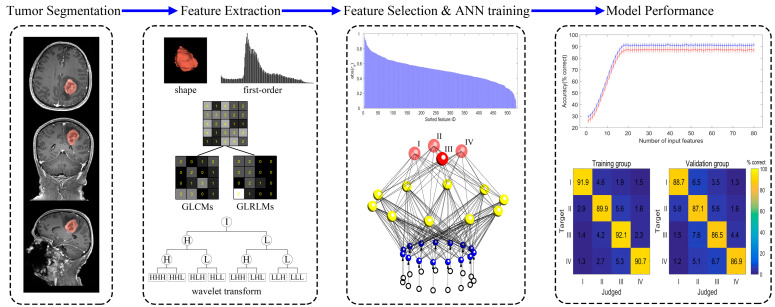
The methodology flowchart of this study. Tumor segmentation was performed on contrast-enhanced T_1_-weighted images. An experienced neuroradiologist delineated the tumor areas on the MRI slices. Radiomics features to quantify tumor signal intensity, shape, and texture were extracted from original MR data. Spearman’s rank correlation analysis was used to assess the correlation between radiomics features and the glioma grades, and the degree of correlation was sorted in descending order to further define the input features of the ANN model (The blue line and the red line represent the training set and the validation set, respectively). The diagnostic performance of the model was assessed by the confusion matrix, which gives the detailed errors of this four-grade classification task.

Radiomics features were extracted using Pyradiomics in Python (version 3.7, https://www.python.org/)(accessed on 20 August 2020). A total of 530 radiomics features were extracted for each patient with Pyradiomics (https://pyradiomics.readthedocs.io/en/latest/) (accessed on 20 August 2020), including both original radiomics features and wavelet-transformed features. The original image features consisted of 18 first-order intensity statistics, 8 shape features, 24 features from gray-level co-occurrence matrices (GLCMs), and 16 features from gray-level run-length matrices (GLRLMs). There were 8 sets of wavelet-transformed features, each set containing 18 first-order intensity statistics, 24 GLCMs, and 16 GLRLMs [22,23]. To select the features most relevant to glioma grades, we conducted Spearman’s rank correlation analysis between the pathological grades and each of the radiomics features with an ICC ≥ 0.75. Spearman’s rank correlation coefficients for each feature were then sorted in descending order. Starting with the first feature, the features were sequentially and accumulatively provided to the ANN as inputs (i.e., the first feature in the first iteration, the first and second features in the second iteration, the first, second, and third features in the third iteration, and so on). The diagnostic accuracy of the ANN model was assessed for each iteration until a performance plateau was reached. The input features corresponding to the beginning of the plateau were ultimately chosen as the input features for the ANN. The workflow of the methodology for this study is briefly illustrated in Figure 1.

### 2.5. Development of ANN

The ANN used in this study to classify the pathological grades was a feedforward back-propagation multilayer perceptron [24] implemented in Matlab (Matlab R2018b) using the Neural Network Toolbox. The ANN consisted of an input layer, a hidden layer, and an output layer. For this study, the number of nodes in the input layer equaled the number of finally chosen input features, while the number of nodes in the output layer was four, corresponding to the four pathological glioma grades (grades I, II, III, and IV). The number of nodes in the hidden layer was based on the formula Nh = (Ni + No)1/2 + α [25]; here, Nh represents the number of nodes in the hidden layer, Ni and No denote the number of nodes in the input and output layers, respectively, and α is a constant ranging from 1 to 10 [26]. Based on a previous study by our team, we set α to 5. The transfer functions of the hidden and output layers adopted the default settings of “tansig” and “purelin”, respectively, in the toolbox.

**Figure 2:**
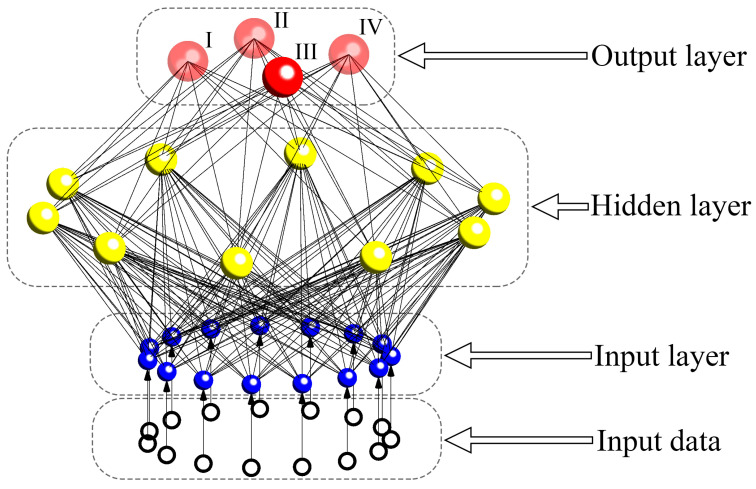
The architecture of the ANN model. The number of nodes in the hidden layer and the input layer is not necessarily the same as that displayed in this diagram but is assigned according to the result of the experiment. The four output nodes represent grades I–IV of glioma, respectively. The input data are the values of radiomics features for each sample.

As a validation set should ideally constitute 25–40% of the training set to estimate the model’s true diagnostic performance [27], the ratio of the training and validation cohorts in this study was set to 3:1 (33.3% of the training set), with 204 randomly selected patients comprising the training set and the remaining 68 patients forming the validation set. The architecture of the ANN model is briefly outlined in Figure 2. The network’s training procedure was set to terminate when the number of training iterations reached 200 or the sum of squared errors (SSE) became less than 0.01 [24].

### 2.6. Statistical Analysis

All statistical analyses were conducted in Matlab (Matlab R2021b). As the task involved a four-grade classification rather than a conventional binary classification, no receiver operating characteristic (ROC) curves were obtained. Instead, a diagnostic confusion matrix was adopted as an intuitive representation of the model’s diagnostic performance. The procedure of randomly splitting (with a constant ratio of 3:1) and the subsequent training and validation were then repeated one hundred times. In this manner, each splitting training validation iteration is referred to as a repetition. The average accuracy score across the one hundred repetitions was obtained as the model’s result. 

## 3. Results

### 3.1. Patient Characteristics

Our study encompassed 362 patients. Patient ages ranged from 16.5 to 71.5 years, with a median age of 44.8 years (interquartile range: 31.7–59.6 years). Table 1 provides a summary of the demographic composition of the patient cohort for reference. It is important to note that our study concentrated on the contribution of radiomics features to the four-grade glioma classification based on the ANN model; demographic factors such as age and gender were not considered in the analysis of this study.

### 3.2. Radiomics Feature Selection

**Figure 3:**
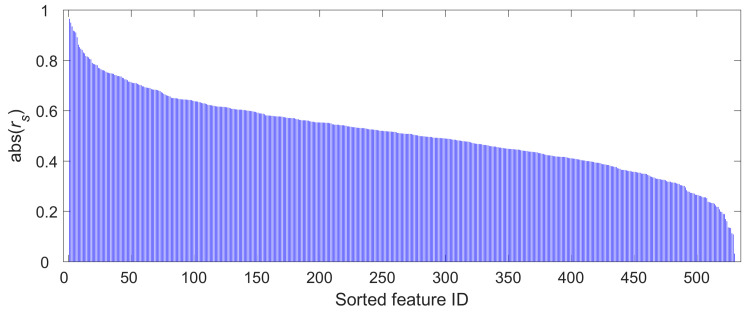
Stemplot of Spearman’s rank correlation coefficients (rs) of these 530 radiomics features. The vertical axis represents the absolute values of rs. These absolute values were sorted in descending order, and the corresponding radiomics features were fed into the ANN model sequentially and accumulatively to assess the diagnostic performance of the model.

**Figure 4:**
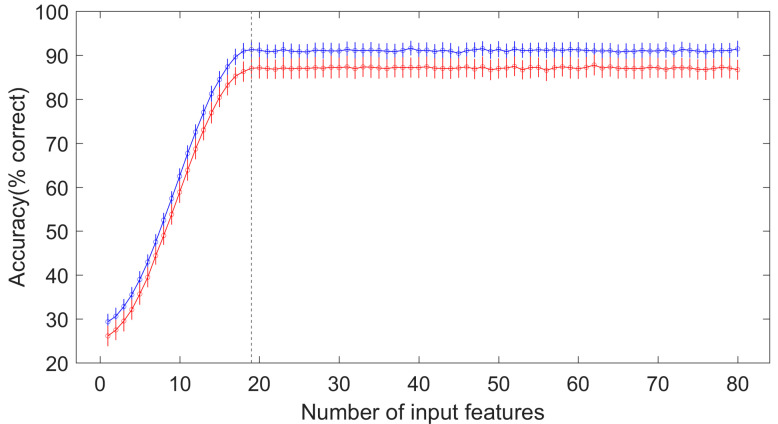
The diagnostic accuracy of the ANN model for each iteration. Each iteration includes the procedure of one hundred times of random group splitting and the following training and validation. The mean accuracy of these one hundred repetitions is represented by the hollow circle, and the standard deviation is depicted by the short vertical bar above the circle. The gray vertical dashed line represents the location where the performance of the model reached a plateau, with nineteen radiomics features as inputs at this point. The blue line and the red line represent the training set and the validation set, respectively.

The 530 extracted radiomics features were initially evaluated using the ICC with a 0.75 criterion. All features had high ICCs, ranging from 0.8157 to 0.9836. Consequently, no radiomics features were excluded by the ICC examination, and all features underwent Spearman’s rank correlation analysis. Figure 3 presents the correlation coefficients (rs) of each radiomics feature in descending order. As mentioned in Materials and Methods, the sorted features were fed into the ANN model sequentially and accumulatively. The performances of the ANN for each iteration (here, an iteration refers to a feed to the ANN, either with one feature for the first iteration or with multiple features for later iterations) are illustrated in a line plot in Figure 4. This clearly shows that the diagnostic performance of the model plateaued after the first nineteen radiomics features were input into the model, both for the training and validation sets. The mean diagnostic accuracies at this point were 0.9128 (95% CI: 0.9094–0.9162) and 0.8704 (95% CI: 0.8657–0.8750) for the training and validation sets, respectively. Therefore, these nineteen radiomics features were used as input for the final ANN model. Table 2 displays the names and corresponding rs values of these nineteen features. It can also be inferred from Figure 4 that the ANN model had a relatively stable performance across the one hundred repetitions at this nineteen-feature point or other points. The standard deviations for this nineteen-feature point (see the location of the vertical gray dashed line in Figure 4) were 0.0173 and 0.0237 for the training and validation sets, respectively. The corresponding coefficients of variation (CVs) were 0.0190 (0.0173/0.9128) and 0.0272 (0.0237/0.8704), respectively.

### 3.3. The Diagnostic Performance of the ANN Model

**Figure 5:**
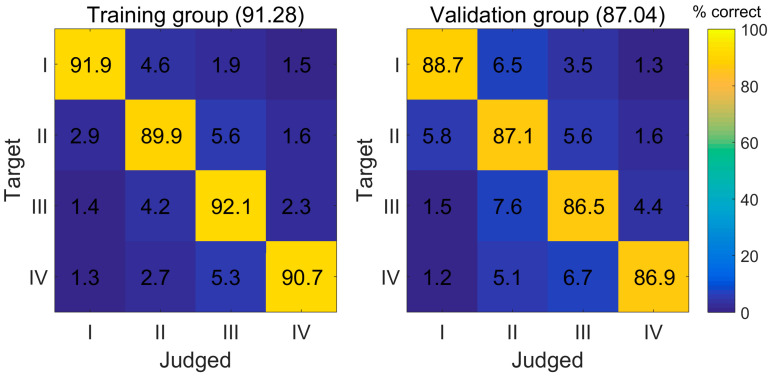
The diagnostic confusion matrices for the training group (left panel) and validation group (right panel). The rows represent the target grades of glioma, and the columns represent the judged grades. The value in each cell indicates the probability of a target grade being classified as grade I, II, III, or IV, respectively. Taking the training group as an example, when the target (real) grade was grade I, the ANN model would have a 91.9% probability of classifying it as grade I and would have a 4.6%, 1.9%, and 1.5% probability of classifying it as grade II, III, and IV, respectively. The colors of the cells are a reflection of the probabilities, with the color bar standing on the right serving as a reference. Note: the value in the parentheses next to the title indicates the averaged overall diagnostic accuracy of the model.

Figure 5 displays the diagnostic confusion matrices of the ANN model, illustrating the detailed classification probabilities for each specific grade of glioma. For a given target grade, the ANN model consistently identifies the target grade with a considerably higher probability than the non-target grades, for both the training and validation sets. It was also observed that the further a grade is from the target, the less likely it is to be identified by the ANN model. In terms of specific values, the diagnostic accuracies for grade I, II, III, and IV gliomas were 91.9%, 89.9%, 92.1%, and 90.7%, respectively, for the training set, and 88.7%, 87.1%, 86.5%, and 86.9%, respectively, for the validation set. The average overall diagnostic accuracies of the ANN for the training and validation sets were 91.28% and 87.04%, respectively.

## 4. Discussion

While glioma diagnosis primarily relies on molecular fingerprinting, the classification of gliomas into grades 1 through 4 remains prevalent in clinical practice, particularly for patients diagnosed as the not otherwise specified (NOS) type. This classification serves as a crucial reference for treatment protocols, adjuvant chemoradiotherapy strategies, response monitoring, and follow-up plans. As reported in the literature, conventional MRI demonstrates high diagnostic value for certain gliomas such as classic diffuse intrinsic pontine glioma (DIPG) [28]. However, it has certain limitations in the diagnosis and grading of atypical gliomas. More importantly, the interpretation of conventional MRI findings carries inherent subjectivity due to its heavy reliance on the expertise of neuroradiologists. To our knowledge, most previous studies on preoperative glioma grading focused on two-grade classification (low grade and high grade), with no reported four-grade classification approach based on radiomics. To address this gap, we employed an artificial neural network model to noninvasively predict glioma grading using radiomics features extracted from post-contrast-enhanced MR images, achieving satisfactory classification results. Our study broadens the limited yet promising evidence supporting the preoperative diagnostic potential of radiomic features derived from post-contrast-enhanced MR images for predicting glioma grades (four-grade classification).

The artificial neural network model, a sophisticated computational model founded on the nonlinear processing of neurons (nodes), has been demonstrated to be an effective diagnostic classification and prediction approach [29,30,31,32,33,34]. The classic application of artificial neural network models for four-grade classification is four-tone recognition. Selecting input variables for the neural network model poses a challenge in such research. In our study, all variables were analyzed in relation to the outcome through univariate analysis, with smaller *p* values indicating greater correlation with the outcome. Features were then selected sequentially based on *p* values, resulting in a total of 20 radiomics features. Notably, most of the selected features in the optimal subset for grading were the gray-level run-length matrix (GLRLM) and gray-level co-occurrence matrix (GLCM). The GLRLM and GLCM represent voxel-based changes in grayscale and can reflect the spatial heterogeneity and complexity of tumors. To some extent, tumor heterogeneity indirectly indicates the degree of malignancy. Although the underlying biological mechanism relating these radiomics features to glioma pathological grading remains unclear, our findings align with those presented in previous studies [35,36]. Our model also achieved favorable classification results in the validation set. As a preliminary attempt, our results demonstrate that the artificial neural network model based on radiomics features extracted from post-contrast-enhanced MR images is stable and reliable, providing a reference for similar studies in the future.

Models based on radiomics features extracted from different sequences exhibit varying predictive performance. Consequently, numerous studies have investigated glioma grading prediction using radiomics features extracted from distinct sequences [37,38,39,40]. For instance, Su et al. [41] proposed a CNN model to predict glioma grading based on multi-modal MRI data, finding that radiomics extracted from post-contrast-enhanced MR images demonstrated the highest grading efficacy. When differentiating glioma subtypes, the best area under the curve (AUC) was 0.896 for grades II–III and 0.881 for grades III–IV. Lin et al. [20] also discovered that among single sequence models, post-contrast-enhanced T_1_WI data yielded optimal grading efficacy. In Tian’s study [21], radiomics features derived from conventional anatomic MRI, particularly enhancement T_1_WI data, achieved relatively high predictive accuracy for distinguishing between grade II and III gliomas. Vidyadharan et al. [42] utilized free water elimination diffusion tensor imaging (FWE-DTI) metrics to classify low-grade gliomas (LGGs) and high-grade gliomas (HGGs), achieving satisfactory classification outcomes. Yu et al. [43] employed a 3D UNet model for differentiating between LGGs and HGGs, where the best AUC reached 0.890. In contrast to these research projects, our study provides a more granular glioma classification (grades I–IV), offering enhanced clinical relevance for practical applications.

Compared to these previous studies, our study directly extracts radiomics features from contrast-enhanced T_1_WI images to predict the pathological grade of glioma patients. Conventionally, different glioma grades often exhibit varying post-contrast enhancement degrees and post-contrast patterns. Notably, tumor enhancement is closely related to blood–brain barrier disruption and tumor angiogenesis, which are crucial factors affecting tumor pathological grading. Therefore, extracting radiomics features based on enhancement T_1_WI images is scientifically and logically sound. Additionally, a single sequence radiomics-based model can reduce the heavy workload caused by multiple sequence delineation while maintaining the same classification effect. Furthermore, enhancement T_1_WI is a conventional sequence, which is simple, convenient, and cost-effective, with better clinical feasibility.

Our study also has some limitations. Firstly, it was a single-center retrospective study; thus, it lacks the external validation that would be attained when using multi-center, large-sample data. This may affect the generalization and universality of our model. Secondly, our data sample size is relatively small for an artificial intelligence study, especially given the severe class imbalance in the distribution of samples in this study. Moreover, we did not consider tumor molecular information, as only a small subset of cases had molecular data on file. Although our findings are promising, they require confirmation in larger sample size and external validation studies in the future. Thirdly, we did not explore the diagnostic efficacy of other conventional single sequences such as T_2_WI, diffusion-weighted imaging (DWI), and FLAIR, which warrant investigation in future studies.

## 5. Conclusions

In response to the prevalent focus on binary classification (LGG and HGG) in current imaging studies for glioma grading, we innovatively propose an ANN model based on radiomic features derived from contrast-enhanced T_1_WI images. This framework seamlessly integrates radiomic features extracted from post-contrast MRI with an ANN architecture to achieve a granular four-grade classification (grades I–IV) of gliomas. Compared to existing glioma classification methods, this technique demonstrates significantly improved grading accuracy. It offers a noninvasive method for preoperative prediction of glioma grading, boasting favorable predictive accuracy and stability. This approach could facilitate clinical decision-making regarding the treatment of patients with various grades of glioma. With the continuous advancement of artificial intelligence (AI) and medical imaging big data, the application of radiomics in glioma research is poised for transformative breakthroughs. Our future work will implement multi-omics approaches, including radio genomics, to achieve comprehensive discrimination, precise grading, and prognostic prediction of gliomas. This integrated strategy aims to empower neurosurgeons with data-driven tools for personalized clinical management of glioma patients.

## Figures and Tables

**Table 1:** Clinical characteristics and glioma grades.

	WHO I	WHO II	WHO III	WHO IV
Number	23	139	81	119
Age (years)	40.8 (16.5–54.7)	43.5 (19.4–60.5)	49.7 (31.7–66.8)	52.2 (29.8–71.5)
Gender				
Male	15	82	46	66
Female	8	57	35	53

Note: the column “Age” presents the mean age in years and the age range (in parenthesis) of that group.

**Table 2:** Selected radiomics features and Spearman correlation coefficients (rs).

Selected Features	|rs|
Wavelet–HLL–First order–Skewness	0.9648
Original–GLRLM–Run-length non-uniformity normalized	0.9494
Wavelet–LHH–GLCM–Joint energy	0.9342
Wavelet–LHL–First order–Maximum	0.9171
Wavelet–HHH–First order–Kurtosis	0.9147
Wavelet–HHH–First order–Median	0.9104
Wavelet–LLH–First order–Skewness	0.8915
Original–GLRLM–Gray-level non-uniformity	0.8619
Wavelet–LLL–GLCM–MCC	0.852
Wavelet–HLH–GLRLM–Run entropy	0.8454
Wavelet–HLH–First order–Kurtosis	0.841
Original–GLCM–Inverse variance	0.8309
Wavelet–LHH–GLCM–Idn	0.827
Original–GLRLM–LRLGLE	0.817
Wavelet–HHH–GLCM–Difference variance	0.8146
Wavelet–LLH–GLCM–Maximum probability	0.8118
Wavelet–LHL–GLRLM–LRLGLE	0.8056
Wavelet–HHL–GLCM–Inverse variance	0.804
Wavelet–LLL–First order–Kurtosis	0.7896

Note: |rs| means the absolute value of rs. These radiomics features sequentially correspond to the first nineteen radiomics features in Figure 3. Abbreviations: GLCM, gray-level co-occurrence matrix; GLRLM, gray-level run-length matrix; H, high-pass filtering; L, low-pass filtering; LRLGLE, long-run low-gray-level emphasis; MCC, maximal correlation coefficient; Idn, inverse difference normalized.

## Data Availability

The data supporting the findings of this study can be obtained from the corresponding author upon reasonable request.

## Abbreviations

The following abbreviations are used in this manuscript:

MRI	Magnetic resonance imaging
ANN	Artificial neural network
CNS	Central nervous system
CE-T_1_WI	Contrast-enhanced T1-weighted images
T_1_WI	T1-weighted images
T_2_WI	T2-weighted images
FLAIR	Fluid-attenuated inversion recovery
ICC	Intra-observer correlation coefficient

## References

[1] Lapointe S., Perry A., Butowski N.A. (2018). Primary brain tumours in adults. Lancet.

[2] Jiang T., Nam D.H., Ram Z., Poon W.S., Wang J., Boldbaatar D., Mao Y., Ma W., Mao Q., You Y. (2021). Clinical practice guidelines for the management of adult diffuse gliomas. Cancer Lett..

[3] Weller M., van den Bent M., Preusser M., Le Rhun E., Tonn J.C., Minniti G., Bendszus M., Balana C., Chinot O., Dirven L. (2021). EANO guidelines on the diagnosis and treatment of diffuse gliomas of adulthood. Nat. Rev. Clin. Oncol..

[4] Louis D.N., Perry A., Wesseling P., Brat D.J., Cree I.A., Figarella-Branger D., Hawkins C., Ng H.K., Pfister S.M., Reifenberger G. (2021). The 2021 WHO Classification of Tumors of the Central Nervous System: A summary. Neuro Oncol..

[5] Lombardi G., Barresi V., Castellano A., Tabouret E., Pasqualetti F., Salvalaggio A., Cerretti G., Caccese M., Padovan M., Zagonel V. (2020). Clinical Management of Diffuse Low-Grade Gliomas. Cancers.

[6] Nabors L.B., Portnow J., Ahluwalia M., Baehring J., Brem H., Brem S., Butowski N., Campian J.L., Clark S.W., Fabiano A.J. (2020). Central Nervous System Cancers, Version 3.2020, NCCN Clinical Practice Guidelines in Oncology. J. Natl. Compr. Canc Netw..

[7] Patel K.S., Carter B.S., Chen C.C. (2018). Role of Biopsies in the Management of Intracranial Gliomas. Prog. Neurol. Surg..

[8] D’Angelo L., Armocida D., Sampirisi L., Paglia F., Berra L.V., Santoro A. (2020). Role of endoscopic surgical biopsy in diagnoses of intraventricular/periventricular tumors: Review of literature including a monocentric case series. Acta Neurol. Belg..

[9] Lara-Almunia M., Hernandez-Vicente J. (2021). Related factors with diagnostic yield and intracranial hemorrhagic complications in frame-based stereotactic biopsy. Review. Neurocirugia.

[10] Thust S.C., Heiland S., Falini A., Jager H.R., Waldman A.D., Sundgren P.C., Godi C., Katsaros V.K., Ramos A., Bargallo N. (2018). Glioma imaging in Europe: A survey of 220 centres and recommendations for best clinical practice. Eur. Radiol..

[11] Habib A., Jovanovich N., Hoppe M., Ak M., Mamindla P., RColen R., Zinn P.O. (2021). MRI-Based Radiomics and Radiogenomics in the Management of Low-Grade Gliomas: Evaluating the Evidence for a Paradigm Shift. J. Clin. Med..

[12] Verburg N., de Witt Hamer P.C. (2021). State-of-the-art imaging for glioma surgery. Neurosurg. Rev..

[13] Tagliafico A.S., Piana M., Schenone D., Lai R., Massone A.M., Houssami N. (2020). Overview of radiomics in breast cancer diagnosis and prognostication. Breast.

[14] Lohmann P., Galldiks N., Kocher M., Heinzel A., Filss C.P., Stegmayr C., Mottaghy F.M., Fink G.R., Jon Shah N., Langen K.J. (2021). Radiomics in neuro-oncology: Basics, workflow, and applications. Methods.

[15] Liu L., Yi X., Lu C., Qi L., Zhang Y., Li M., Xiao Q., Wang C., Zhang L., Pang Y. (2020). Applications of radiomics in genitourinary tumors. Am. J. Cancer Res..

[16] Mao Y., Wang J., Zhu Y., Chen J., Mao L., Kong W., Qiu Y., Wu X., Guan Y., He J. (2022). Gd-EOB-DTPA-enhanced MRI radiomic features for predicting histological grade of hepatocellular carcinoma. Hepatobiliary Surg. Nutr..

[17] Zhang L., Zhu X., Farewik E.M.G., Wang R. (2022). Ankle Joint Torque Prediction Using an NMS Solver Informed-ANN Model and Transfer Learning. IEEE J. Biomed. Health Inform..

[18] Fan H., Su P., Huang J., Liu P., Lu H. (2021). Multi-band MR fingerprinting (MRF) ASL imaging using artificial-neural-network trained with high-fidelity experimental data. Magn. Reson. Med..

[19] Ding J., Zhao R., Qiu Q., Chen J., Duan J., Cao X., Yin Y. (2022). Developing and validating a deep learning and radiomic model for glioma grading using multiplanar reconstructed magnetic resonance contrast-enhanced T1-weighted imaging: A robust, multi-institutional study. Quant. Imaging Med. Surg..

[20] Lin K., Cidan W., Qi Y., Wang X. (2022). Glioma grading prediction using multiparametric magnetic resonance imaging-based radiomics combined with proton magnetic resonance spectroscopy and diffusion tensor imaging. Med. Phys..

[21] Tian Q., Yan L.F., Zhang X., Zhang X., Hu Y.C., Han Y., Liu Z.C., Nan H.Y., Sun Q., Sun Y.Z. (2018). Radiomics strategy for glioma grading using texture features from multiparametric MRI. J. Magn. Reson. Imaging.

[22] Chen J., Lu S.H., Mao Y.T., Tan L., Li G., Gao Y., Tan P.Q., Huang D.H., Zhang X., Qiu Y.Z. (2022). An MRI-based radiomics-clinical nomogram for the overall survival prediction in patients with hypopharyngeal squamous cell carcinoma: A multi-cohort study. Eur. Radiol..

[23] Gao Y., Mao Y.T., Lu S.H., Tan L., Li G., Chen J., Huang D.H., Zhang X., Qiu Y.Z., Liu Y. (2021). Magnetic resonance imaging-based radiogenomics analysis for predicting prognosis and gene expression profile in advanced nasopharyngeal carcinoma. Head Neck.

[24] Mou Z.W., Ye W.J., Chang C.C., Mao Y.T. (2020). The Application Analysis of Neural Network Techniques on Lexical Tone Rehabilitation of Mandarin-Speaking Patients with Post-Stroke Dysarthria. IEEE Access.

[25] Lin B., Lin G.T., Liu X.Y., Ma J.S., Wang X.C., Lin F.Y., Hu L.F. (2015). Application of back-propagation artificial neural network and curve estimation in pharmacokinetics of losartan in rabbit. Int. J. Clin. Exp. Med..

[26] Ma J.S., Cai J.Z., Lin G.Y., Chen H.L., Wang X.Q., Wang X.C., Hu L.F. (2014). Development of LC-MS determination method and back-propagation ANN pharmacokinetic model of corynoxeine in rat. J. Chromatogr. B.

[27] Papanikolaou N., Matos C., Koh D.M. (2020). How to develop a meaningful radiomic signature for clinical use in oncologic patients. Cancer Imaging.

[28] Lo Greco M.C., Marano G., La Rocca M., Acquaviva G., Milazzotto R. (2025). Latest Advancements in the Management of H3K27M-Mutant Diffuse Intrinsic Pontine Glioma: A Narrative Review. Cancers.

[29] Asteris P.G., Gavriilaki E., Touloumenidou T., Koravou E.E., Koutra M., Papayanni P.G., Pouleres A., Karali V., Lemonis M.E., Mamou A. (2022). Genetic prediction of ICU hospitalization and mortality in COVID-19 patients using artificial neural networks. J. Cell Mol. Med..

[30] Bede P., Murad A., Hardiman O. (2022). Pathological neural networks and artificial neural networks in ALS: Diagnostic classification based on pathognomonic neuroimaging features. J. Neurol..

[31] Zhou M., Ji J., Xie N., Chen D. (2022). Prediction of birth weight in pregnancy with gestational diabetes mellitus using an artificial neural network. J. Zhejiang Univ. Sci. B.

[32] Mai R.Y., Lu H.Z., Bai T., Liang R., Lin Y., Ma L., Xiang B.D., Wu G.B., Li L.Q., Ye J.Z. (2020). Artificial neural network model for preoperative prediction of severe liver failure after hemihepatectomy in patients with hepatocellular carcinoma. Surgery.

[33] Pathak P., Panday S.B., Ahn J. (2022). Artificial neural network model effectively estimates muscle and fat mass using simple demographic and anthropometric measures. Clin. Nutr..

[34] Gutta S., Acharya J., Shiroishi M.S., Hwang D., Nayak K.S. (2021). Improved Glioma Grading Using Deep Convolutional Neural Networks. AJNR Am. J. Neuroradiol..

[35] Tabatabaei M., Razaei A., Sarrami A.H., Saadatpour Z., Singhal A., Sotoudeh H. (2021). Current Status and Quality of Machine Learning-Based Radiomics Studies for Glioma Grading: A Systematic Review. Oncology.

[36] Zhou H., Xu R., Mei H., Zhang L., Yu Q., Liu R., Fan B. (2022). Application of Enhanced T1WI of MRI Radiomics in Glioma Grading. Int. J. Clin. Pract..

[37] Li G., Li L., Li Y., Qian Z., Wu F., He Y., Jiang H., Li R., Wang D., Zhai Y. (2022). An MRI radiomics approach to predict survival and tumour-infiltrating macrophages in gliomas. Brain.

[38] Choi Y.S., Bae S., Chang J.H., Kang S.G., Kim S.H., Kim J., Rim T.H., Choi S.H., Jain R., Lee S.K. (2021). Fully automated hybrid approach to predict the IDH mutation status of gliomas via deep learning and radiomics. Neuro-Oncology.

[39] Yan J., Zhang B., Zhang S., Cheng J., Liu X., Wang W., Dong Y., Zhang L., Mo X., Chen Q. (2021). Quantitative MRI-based radiomics for noninvasively predicting molecular subtypes and survival in glioma patients. NPJ Precis. Oncol..

[40] Zhuo Z., Qu L., Zhang P., Duan Y., Cheng D., Xu X., Sun T., Ding J., Xie C., Liu X. (2021). Prediction of H3K27M-mutant brainstem glioma by amide proton transfer-weighted imaging and its derived radiomics. Eur. J. Nucl. Med. Mol. Imaging.

[41] Su C., Jiang J., Zhang S., Shi J., Xu K., Shen N., Zhang J., Li L., Zhao L., Zhang J. (2019). Radiomics based on multicontrast MRI can precisely differentiate among glioma subtypes and predict tumour-proliferative behaviour. Eur. Radiol..

[42] Vidyadharan S., Rao B.P., Yogeeswari P., Kesavadas C., Rajagopalan V. (2024). Accurate low and high grade glioma classification using free water eliminated diffusion tensor metrics and ensemble machine learning. Sci. Rep..

[43] Yu X., Wu Y., Bai Y., Han H., Chen L., Gao H., Wei H., Wang M. (2022). A lightweight 3D UNet model for glioma grading. Phys. Med. Biol..

